# The role of extracellular matrix in mouse and human corneal neovascularization

**DOI:** 10.1038/s41598-019-50718-8

**Published:** 2019-10-03

**Authors:** M. Barbariga, F. Vallone, E. Mosca, F. Bignami, C. Magagnotti, P. Fonteyne, F. Chiappori, L. Milanesi, P. Rama, A. Andolfo, G. Ferrari

**Affiliations:** 1 Cornea and Ocular Surface Disease Unit, Eye Repair Lab, IRCCS San Raffaele Scientific Institute, Milan, Italy; 2 ProMiFa, Protein Microsequencing Facility, IRCCS-San Raffaele Scientific Institute, Milan, Italy; 3 Institute of Biomedical Technologies, National Research Council, Segrate, MI Italy

**Keywords:** Protein-protein interaction networks, Preclinical research, Molecular medicine

## Abstract

Corneal neo-vascularization (CNV) is a highly prevalent medical condition which impairs visual acuity. The role of specific proteins in modulating CNV has been extensively reported, although no studies have described the entire human proteome in CNV corneas. In this paper, we performed a proteomic analysis of vascularized *vs* healthy corneal stroma, in a CNV mouse model and in CNV-affected patients, with a specific focus on extracellular matrix (ECM) proteins. We identified and quantified 2315 murine proteins, 691 human proteins and validated 5 proteins which are differentially expressed in vascularized samples and conserved in mice and humans: tenascin-C and fibronectin-1 were upregulated, while decorin, lumican and collagen-VI were downregulated in CNV samples. Interestingly, among CNV patients, those affected with Acanthamoeba keratitis showed the highest levels of fibronectin-1 and tenascin-C, suggesting a specific role of these two proteins in Acanthamoeba driven corneal CNV. On a broader picture, our findings support the hypothesis that the corneal stroma in CNV samples is disorganized and less compact. We are confident that the dissection of the human corneal proteome may shed new light on the complex pathophysiology of human CNV, and finally lead to improved treatments.

## Introduction

Proper vision requires corneal avascularity^1^. This is a finely regulated process, and relies on constitutive expression of anti-angiogenic molecules^2,3^, while pro-angiogenic factors are inhibited^4,5^.

Corneal neo-vascularization (CNV) is the second cause of blindness worldwide^6^ even if, being a consequence of several primary pathologies, its prevalence is still under debate^7^. In any case, it is the result of a multitude of ocular and systemic diseases, which interrupt the corneal angiogenic privilege. Clinical conditions causing CNV include trauma, infections, autoimmunity and allergies^8–11^. When CNV has developed and vision has been impaired, corneal transplantation can be attempted to restore corneal transparency. Pre-existing CNV, however, significantly reduces the survival of the corneal graft^12^. In summary, CNV is a significant clinical problem and a better understanding of its pathophysiology is needed to develop effective treatments.

The corneal proteome has been studied during angiogenesis, although limitedly to: animal models, during the acute phase of CNV, and not in the isolated corneal stroma. Previous works^13,14^ have elucidated the role of soluble factors in CNV, including VEGFs^15^, thrombospondin-1 and -2 (THBS-1 and -2)^16^, metalloproteases (MMPs)^17^, basic fibroblast growth factor (bFGF)^4^ and pigment epithelium-derived factor (PEDF)^14^.

The role of the extracellular matrix (ECM) in CNV, instead, is less studied, and the contribution of specific ECM proteins to human CNV remains unclear. A key role for ECM proteins in CNV is suggested by many observations. First, they can modulate the activity of both pro-angiogenic molecules and anti-angiogenic factors^18–20^. Second, ECM could control corneal biomechanics, by interacting with MMPs and promote angiogenesis^17,21^. Among mechanical properties of the cornea, tissue rigidity^22^ is fundamental to control endothelial cell invasion and the development of neovessels, as it occurs in tumors^23^. Finally, ECM degradation can release soluble factors which exacerbate inflammation and vessel growth^24^.

We have previously published that intra-stromal application of sutures in mice is a robust and highly reproducible CNV model, which permits imaging and exact quantification of both blood and lymphangiogenesis^25^. In addition, this model is well suited to study not only the growth phase of CNV, but also its regression, which approximates this model to the clinical course of CNV.

In this paper, we compared the protein expression profile between acute and chronically vascularized *vs* healthy corneal stroma in mice and humans. We specifically focused on ECM proteins, in order to elucidate their contribution to CNV pathophysiology and with the final aim of finding novel therapeutic targets.

## Material and Methods

### Animals

**Figure 1:**
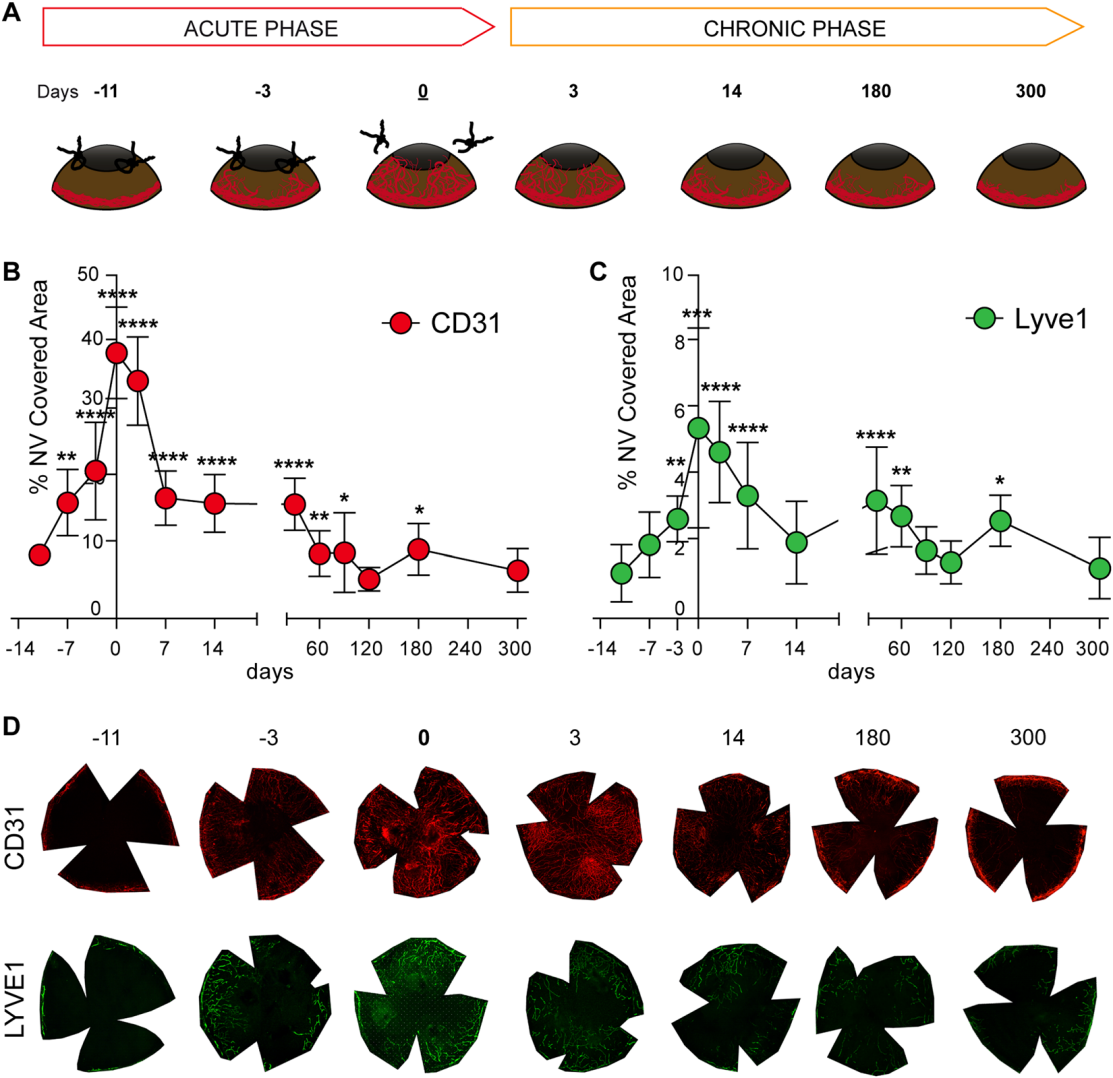
Suture induced blood and lymphatic vessel growth in the cornea. (**A**) Cartoon showing the CNV-inducing mouse model we designed. Graphs showing the growth rate of blood (panel B) and lymphatic (panel C) vessels, measured in acute and chronic phases. (**D**) Representative pictures of blood and lymphatic vascularization over time, in red and green respectively. Asterisks in panel B and C represent the difference between suture and controls, graphs represent mean values ± SEM; statistical analysis measured by One-Way ANOVA analysis, following Tukey multiple comparison tests (**p < 0.01, ****p < 0.0001).

Male, 8 week-old BALB/C mice (Charles-River) were used for all experiments (116 mice in total, 30 as control and 86 sutured). All experimental protocols were approved by the Animal Care and Use Committee of the IRCCS San Raffaele Scientific Institute, in accordance with the ARVO Statement for the Use of Animals in Ophthalmic and Vision Research. Animals did not show evident signs of distress during the course of the study, and their weight remained normal. Animals were allowed to acclimatize in their environment for 1 week before experimentation and each animal was deeply anesthetized with intraperitoneal injection of Tribromoethanol (250 mg/kg) before all surgical procedures. To induce CNV, mice were anesthetized and sutures were placed intra-stromally in the corneas, as previously described with some modifications^15^. We used 86 bilaterally sutured-mice: at least 6 corneas per time point for IF analysis (120 in total) and 4 pooled corneas (from 4 different animals) for proteomics analysis (52 in total). Thanks to a 2-mm corneal trephine placed on the cornea and centred on the pupil, three 10.0 nylon sutures were placed intra-stromally 120° apart with knots left unburied. Post-operatively, all animals received a single dose of Carprofen at 5 mg/kg subcutaneously. Sutures were left in place for 14 days (Fig. 1A), during which animals were imaged *in vivo* every day to track vessel formation. This daily imaging procedure is fast and painless for the animals, therefore no anaesthesia was performed. After suture removal, mice were monitored up to 10 months to evaluate the persistence of a chronic vascularization phase. Setting the day of suture removal as time point 0, different groups of mice were sacrificed during acute CNV at −11, −7 and −3 days and during chronic CNV after 3, 7, 14, 30, 60, 90, 120, 180 and 300 days. Carbon dioxide inhalation and subsequent cervical dislocation were applied to euthanize the animals; corneas were then removed with a sharp scalpel under the stereo microscope and placed in PBS.

Non-vascularized corneas from mice (n = 30) provided controls (8 corneas for IF analysis, and 52 age-matched corneas for proteomics analysis, 4 pooled corneas per each time point).

### Murine cornea immunohistochemistry

After microsurgical excision, the corneal stroma and epithelium were separated with 20 mM EDTA in phosphate buffer (PBS) for 30 min at 37 °C. Corneal stroma was then fixed for 15 min in ice-cold acetone, washed with PBS and blocked for 1 h with 2% bovine serum albumin (BSA; Sigma-Aldrich), 5% normal donkey serum (Sigma-Aldrich) in PBS. Tissue was incubated at 4 °C o/n with primary antibodies (rat anti-mouse CD31, BioLegend, and rabbit anti-mouse LYVE1, AbCam) in blocking solution, followed by secondary antibodies in PBS 2 h at RT (AlexaFluor 594 donkey anti-rat and AlexaFluor 488 donkey anti-rabbit, Invitrogen). After washing in PBS, corneas were radially cut and flat mounted on glass slides using the Vectashield mounting medium with 4′,6-Diamidino-2-Phenylindole (DAPI, Vector). Images of CNV were analyzed using an ImageJ-based system to quantify the total area of the cornea covered by vessels.

### Patients

CNV affected patients (n = 19, see Table 1) planning to undergo corneal transplant were selected, after obtaining informed consent and after approval by the San Raffaele Hospital Ethics Committee, by the Cornea and Ocular Surface Unit at the San Raffaele Scientific Institute. CNV was quantified by counting the number of corneal quadrants involved, with a score ranging from 1 (neovessels involving 1 quadrant) to 4 (neovessels involving four quadrants) at the time of corneal transplant. CNV patients in this study had an average CNV score of 3.05 ± 1.17. Fresh corneal buttons obtained from these patients immediately after keratoplasty were collected in Carry-C transport solution (Alchimia) for subsequent proteomics or Western Blot/ELISA analysis. Avascular, keratoconic corneas (n = 27, Table 1) were used as avascular controls. Methods used to obtain human data were performed in accordance with the relevant guidelines and regulations.

**Table 1:** Patient data.

	Number	Age	Sex	Diagnosis	Infection	CNV score	Used For
CONTROLS	#1	67	F	Keratoconus	No	/	MS, WB
	#2	33	F	Keratoconus	No	/	MS, WB
	#3	37	M	Keratoconus	No	/	MS, WB
	#4	42	M	Keratoconus	No	/	MS, WB
	#5	35	M	Keratoconus	No	/	MS, WB
	#6	42	M	Keratoconus	No	/	MS, WB
	#7	40	F	Keratoconus	No	/	MS, WB
	#8	39	M	Keratoconus	No	/	MS, WB
	#9	57	F	Keratoconus	No	/	ELISA
	#10	43	F	Keratoconus	No	/	ELISA
	#11	68	F	Keratoconus	No	/	ELISA
	#12	54	F	Keratoconus	No	/	ELISA
	#13	64	M	Keratoconus	No	/	ELISA
	#14	64	F	Keratoconus	No	/	ELISA
	#15	53	M	Keratoconus	No	/	ELISA
	#16	68	M	Keratoconus	No	/	ELISA
	#17	52	F	Keratoconus	No	/	ELISA
	#18	59	F	Keratoconus	No	/	ELISA
	#19	28	M	Keratoconus	No	/	ELISA
	#20	55	F	Keratoconus	No	/	ELISA
	#21	45	F	Keratoconus	No	/	ELISA
	#22	51	F	Keratoconus	No	/	ELISA
	#23	22	M	Keratoconus	No	/	ELISA
	#24	18	M	Keratoconus	No	/	ELISA
	#25	23	M	Keratoconus	No	/	ELISA
	#26	42	M	Keratoconus	No	/	ELISA
	#27	46	M	Keratoconus	No	/	ELISA
CNV	#28	51	F	Aniridia	No	4	MS, WB
	#29	48	M	Perforating keratoplasty graft rejection	No	4	MS, WB
	#30	69	M	Herpetic keratitis, leucoma	Yes	2	MS, WB
	#31	63	F	Herpetic keratitis, lipid degeneration	Yes	4	MS, WB
	#32	41	M	Contact lens abuse, Lasik	No	2	MS, WB
	#33	48	M	Micotic keratitis, leucoma	Yes	2	MS, WB
	#34	46	F	Allergic conjunctivitis	No	4	MS, WB
	#35	49	M	Perforating keratoplasty graft rejection	No	4	MS, WB
	#36	58	M	Perforating keratoplasty graft rejection	No	2	MS, WB
	#37	62	M	Lens abuse, blefaritis, glaucoma	Yes	4	MS, WB
	#38	40	M	Perforating keratoplasty graft rejection	No	4	ELISA
	#39	57	F	Acanthamoeba keratitis	Yes	4	ELISA
	#40	70	F	Herpetic keratouveitis	Yes	4	ELISA
	#41	27	F	Herpetic keratitis, corneal perforation	Yes	2	ELISA
	#42	63	M	Contact lens abuse	No	1	ELISA
	#43	45	F	Acanthamoeba keratitis	Yes	2	ELISA
	#44	77	F	Perforating keratoplasty graft rejection	Yes	4	ELISA
	#45	69	M	Perforating keratoplasty graft rejection	No	1	ELISA
	#46	32	M	Acanthamoeba keratitis	Yes	4	ELISA

Table showing controls and CNV patients’ demographics. The type of analysis performed on the cornea is reported in the right column.

### Murine and human cornea protein extraction

Murine pooled samples and human corneas were processed according to the following protocol.

Upon incubation in 20 mM EDTA with protease inhibitor cocktail (Sigma-Aldrich) for 40 min at 37 °C, epithelium was removed from the stroma using forceps with the help of a stereomicroscope. Corneal endothelium was not removed from the stroma, since it’s impact on stroma ECM proteomics is negligible^26^. For the proteomic analysis, the stroma was cut in small pieces and put in 100 µl of R3 buffer (urea 5M, thiourea 2M, CHAPS 2% w/v, Zwittergent 2% w/v) plus protease inhibitor cocktail. Samples were subsequently homogenized with a plastic potter and incubated on a rotating shaker for 1 h at RT. After centrifugation (14,000 × g, for 10 min at RT), supernatants were used for proteome analysis.

### Mass Spectrometry and Proteomics analysis

Proteins were quantified using Bradford assay and BSA as standard. Proteins were digested using the FASP (Filter Aided Sample Preparation) protocol^27^. Briefly, 20 µg of total proteins from each sample were diluted to 200 µl with 0.1 M Tris/HCl pH 7.4. Cysteines were reduced with 45 µl of 0.1 M DTE (1,4-dithioerythritol) in Tris/HCl pH 7.4 and incubated at 95 °C for 5 min; then samples were centrifuged on Amicon Ultra-10K at 14,000 × g for 10 min with a solution of urea 8 M. Cysteines were alkylated with 100 µl 0.05 M IAA (Iodoacetamide) in urea 8 M added on the filter and laid at RT for 5 min; samples were centrifuged on Amicon Ultra-10K at 14,000 × g for 10 min with a solution of urea 8 M and then incubated overnight at 37 °C in the presence of trypsin 1:50 (w/w), upon dilution of urea up to 2 M with 50 mM ammonium bicarbonate buffer.

Peptide mixtures were desalted on homemade Stage Tips C18 and injected in a capillary chromatographic system (EASY-nLC™ 1000 Integrated Ultra High Pressure Nano-HPLC System, Proxeon Biosystem) for peptide separations on a 75 µm i.d. × 12 cm reverse phase silica capillary column, packed with 1.9 μm ReproSil-Pur 120 C18-AQ. A 95 min-gradient of eluents A (pure water with 0.1% v/v formic acid) and B (ACN with 0.1% v/v formic acid) was used to achieve separation (from 5% to 50% of B in 88 min, 0.30 μL/min flow rate). MS analyses were performed using a Q-Exactive mass spectrometer (Thermo Scientific). Each sample was analyzed in technical triplicates. Full scan spectra were acquired with resolution set to 70,000 and mass range from m/z 380 to 1800 Da. The ten most intense doubly and triply charged ions were selected to be fragmented (ddMS2). MS/MS spectra were acquired with resolution set to 17,500, NCE set to 25 with an isolation window of 2 m/z. All data were analyzed by MaxQuant software (v. 1.5.2.8)^28^ for label-free protein quantification based on the precursor intensity, using the following search parameters: UniProtKB_complete_proteome_20161130 as database; up to 2 missed cleavages allowed; carbamidomethylation of cysteine as fixed modification; N-terminus-acetylation and Methionine oxidation as variable modifications; ±5 ppm and ±20 ppm for precursor and fragment ions mass tolerance, respectively. The proteins identified in murine and human stroma were subsequently compared with the ECM protein database Matrisome^29^ to confirm the prevalent presence of stromal proteins in the analysed samples.

Human stromal samples were lysed as above reported for the murine samples. The only difference was the digestion with dextranase from Penicillum sp. 1 U/µl (Sigma-Aldrich) in 0.05 M KH2PO4 buffer pH 6 at 37 °C overnight prior to the Bradford assay. In details, human stromal proteins were incubated with dextranase in 1:4.5 (v/v) in order to hydrolyze the dextran, which derived from the medium used for cornea conservation. Proteins were subsequently digested using the FASP protocol and analyzed by mass spectrometry.

### Data Normalization and differential expression assessment

Protein identifiers were converted to Entrez Gene identifiers, and Homologene^30^ was used to pair mouse and human proteins by homology. The mapping yielded proteins with a homologue available in both species and for which we detected valid (not null) proteomic measurements in all samples. Proteomics measurements were normalized using weighted-trimmed mean of M-values^31^ and differential expression was assessed by means of the moderated t test^32^, two approaches initially proposed for gene expression data, but shown to be useful also for proteomics data^33,34^. Nominal p values were corrected using the BH procedure. The R programming environment^35^ and its packages limma^36^ and edgeR^37^ were used to carry out normalization, differential expression and multiple testing correction. Proteins were considered differentially expressed at absolute values of log2 Fold Change (LFC) greater than 0.58, corresponding to FC less than 2/3 or greater than 3/2, and False Discovery Rate (FDR) less than 0.2.

### ELISA

Human corneal stromas were cut in small pieces and resuspended in 100 µl PBS with protease inhibitor cocktail (Sigma-Aldrich). Samples were homogenized with a T110 homogenizer (IKA), 30 sec (power 5, 3 times on ice) and centrifuged at 12,000 × g for 10 min to remove tissue debris. Supernatants were quantified with Bradford protein assay (Thermo scientific, Waltham, Massachusetts, USA and 5 µg of total proteins were analyzed in triplicate with TNC and FN1 Elisa KITs (Cayman), following manufacturer’s instructions.

### Western blot

Five μg of proteins from control or CNV stromas were re-suspended in NuPAGE LDS reducing Sample Buffer (Thermo Fisher), resolved on NuPAGE 4–12% Bis-Tris Protein Gels (Thermo Fisher) and electro-transferred to nitrocellulose membranes (Amersham, Little Chalfont, UK) for Western blot (WB) analysis. Protein transfer was evaluated by red Ponceau S staining (Sigma-Aldrich). Membranes were blocked in a Tris buffered solution (TBS) 5% milk, 0.1% Tween 20 and incubated overnight with primary antibodies: mouse anti-human decorin (MAB143), rabbit anti-human lumican and anti-human collagen-VI α1 (NBP1-87726 and NB120-6588, Novus Biologicals) at 4 °C under gentle shaking. Subsequently, membranes were incubated at RT for 1 h with anti-mouse or anti-rabbit HRP-conjugated secondary antibodies (NA9310V and NA9340V respectively, Ge Healthcare) followed by chemiluminescence reaction performed with ECL detection reagent (Ge Healthcare) and film exposure. The protein band optical density was finally measured with the UVITEC imaging system. Expression of β-actin revealed with an HRP conjugated mouse monoclonal antibody (ab49900, Abcam) was used as loading control.

### Statistics

Corneal vascularization rate over time was analyzed by One-Way ANOVA analysis, following Tukey multiple comparison tests. Unpaired *t*-test was used to evaluate the differences in WB band intensity and ELISA values between control and CNV patients. A p value < 0.05 was considered to be statistically significant. The statistical software GraphPad Prism 5.0 (GraphPad) was used for all analyses. All methods were performed in accordance with the relevant guidelines and regulations.

## Results

### Acute and chronic corneal neovascularization induced by intra-stromal suture

Quantification of the blood and lymphatic vascularization, measured as CD31 and Lyve1 positive area respectively, confirmed a rapid growth of vessels in sutured corneas. The density of blood vessels was significantly higher 7 days after suture implantation (Fig. 1B, day −7, vascularized area 15.3% ± SEM 4.3% p < 0.0001), while lymphatic vessel density reached significance 14 days after surgery (Fig. 1C, day 0, +6.69% ± SEM 2.8%, p < 0.0001). Hem- and lymph-angiogenesis reached the maximum extension just before suture removal (Fig. 1A, day 0, +35.15% ± SEM 6.1%, p < 0.0001 and Fig. 1C +6.69% ± SEM 2.8%, p < 0.0001 for blood and lymphatic vessels respectively). After suture removal, vessel regression was observed; however, sutured corneas remained significantly more vascularized compared to control corneas for the following 2 months (Fig. 1B, day 30, +15.1% ± SEM 3.44%, p < 0.0001 and Fig. 1C, +3.52% ± SEM 1.4%, p < 0.01 for blood and lymphatic vessels respectively). Figure 1D shows representative immunofluorescence pictures of blood (upper pictures) and lymphatic (bottom pictures) vascularization in sutured corneas.

### Murine and human CNV proteomics

Mass spectrometry (MS) analysis of murine corneal stroma throughout the entire vascularization period allowed us to identify and quantify a total number of 2315 proteins. We excluded the 180 days endpoint from our dataset for the proteomic analysis, because the number of the identified proteins was particularly small compared to the other time points. This was probably the consequence of a technical problem occurred during sample preparation. In any case, we did not see any significant difference of CNV extension at this time point by means of immunofluorescence. In human samples (8 controls vs 10 CNV), 691 proteins were detected. Interrogation of the ECM protein-database Matrisome retrieved 115 matrisome-associated proteins (Supplemental Fig. S1A, Supplemental Table S1), in particular 69 ECM regulators, 28 ECM affiliated proteins and 18 secreted factors in mouse corneas. When comparing our data with the core matrisome of ECM, 103 proteins were found, of which 66 belong to ECM glycoproteins, 25 to the collagen family and 12 to ECM proteoglycans (Supplemental Fig. S1B, Supplemental Table S1). In human samples, the comparison identified 74 matrisome-associated proteins with 43 ECM regulator proteins, 14 ECM affiliated proteins and 17 secreted factors (Supplemental Tables S1C, S2); out of the 93 proteins identified as part of the human core matrisome, 60 were ECM glycoproteins, 21 collagens and 12 ECM proteoglycans (Supplemental Fig. S1D, Supplemental Table S2). This data indicates a good coverage of the stroma proteome, both in murine (20%) and human (16%) samples, as expected when compared to the entire species-specific matrisome. Moreover, we could confirm the good technical extraction of ECM proteins by our tissue protein extraction method.

**Figure 2:**
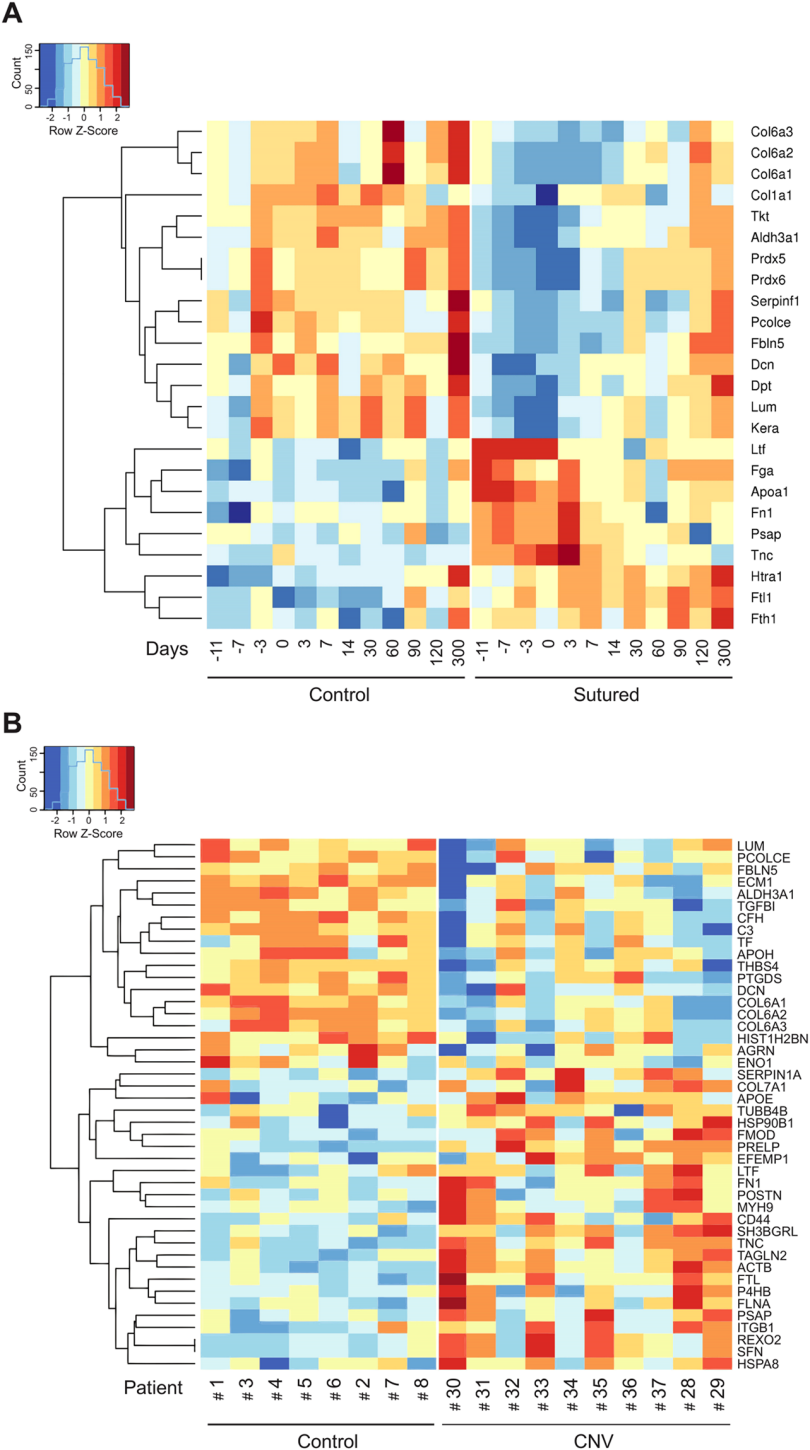
Z-score hierarchical clustering heat map visualization. (**A**) Gene names of the proteins identified by the proteomics analysis as differentially expressed in sutured and control mice at different time points; PRDX5 and PRDX6 are reported, twice, since they refer to two different protein isoforms derived from the same gene; (**B**) Gene names of the proteins differentially expressed in human CNV and control corneal stroma. Only significant proteins (|log2(FC)| >0.58 and FDR <0.2) are reported; colours represent scaled expression values, with blue for low expression and red for the high expression levels.

Among the 110 proteins resulting from homology mapping between the two species (Supplemental Table S3), 26 proteins were differentially expressed (|log2(FC)| >0.58 and FDR <0.2) between sutured animals and controls all over the time (Fig. 2A) and 44 (|log2(FC)| >0.58 and FDR <0.2) between 10 CNV patients and 8 controls (Fig. 2B).

### Comparative analysis of murine and human CNV proteomics

**Figure 3:**
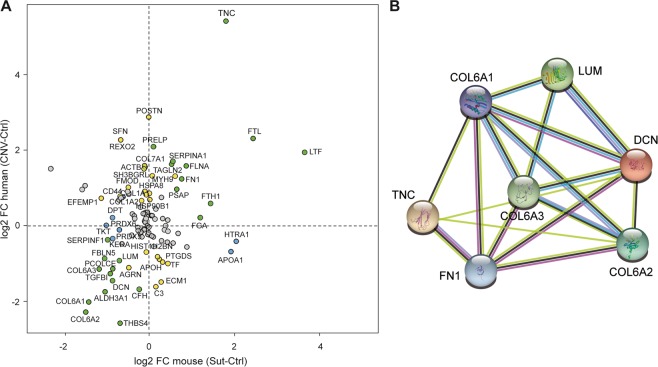
Comparison of mouse and human proteomics data. (**A**) Differential expression (expressed as log2 fold change) of proteins identified in mouse (X axis) and human (Y axis). Yellow dots represent proteins differentially expressed in mouse; blue dots represent proteins differentially expressed in human; green dots represent proteins differentially expressed (Fisher combined probability test on FDRs <0.2) in the same direction in both species. (**B**) String network displaying experimentally determined interactions, interaction from curated database, protein homology, co-expression and co-citation in literature between selected proteins (pink, light blue, purple, black and lime connecting lines respectively). Colored nodes represent proteins added to the search query, regardless of the color.

The comparison of differential expression in human and mouse highlighted several proteins with significant expression changes in the same direction (cases *vs* controls) (Fig. 3A, green dots). Protein–protein interaction network of DEGs was constructed using the Search Tool for the Retrieval of Interacting Genes (STRING, http://string.embl.de/) database^38^. This procedure allowed us to identify 5 ECM proteins in common between mouse and human (STRING network, Fig. 3B), which are strongly related, often cited together in the literature, which interact biologically each other or which are part of the same family. Two of these proteins were upregulated in the murine sutured and human CNV samples (Table 2): tenascin-C (TNC, LFC = +5.40, p = 0.0002 in human; LFC = +1.83, p = 5.86 × 10^−4^ in mouse;) and fibronectin-1 (FN1, LFC = +1.23, p = 0.0270 in human, LFC = +0.78, p = 0.0059 in mouse). The remaining 3 proteins, instead, were downregulated (Table 2): lumican (LUM, LFC = −0.94, p = 0.0250 in human; LFC = −0.71, p = 0.0120 in mouse), decorin (DCN, LFC = −1.46 p = 0.0049 in human; LFC −0.88, p = 0.0112 in mouse), and the three different subunits of collagen-VI (α1, LFC = −2.02, p = 0.0002 in human; LFC = −1.43, p = 0.0047 in mouse. α2, LFC = −2.28, p = 0.0002 in human; LFC = −1.50, p = 0.0028 in mouse. α3, LFC = −1.14, p = 0.0047 in human; LFC = −1.18, p = 0.0074 in mouse.

**Table 2:** ECM proteins with a common expression changes in mouse and human.

	Human	p	FDR	mouse	p	FDR
	LFC			LFC		
TNC	5.40	0.0003	0.0054	1.83	0.0006	0.0320
FN1	1.23	0.0270	0.0940	0.78	0.0059	0.1100
DCN	−1.46	0.0049	0.0370	−0.87	0.0112	0.1500
LUM	−0.94	0.0250	0.0890	−0.71	0.0120	0.1500
COL6A1	−2.02	0.0002	0.0054	−1.43	0.0046	0.1003
COL6A2	−2.28	0.0002	0.0054	−1.50	0.0028	0.0712
COL6A3	−1.14	0.0047	0.0367	−1.18	0.0074	0.1245

Values of log_2_ fold change (LFC), p-value (p) and adjusted p-value (FDR) for each of the proteins with significant expression changes in the same direction (cases vs controls). Human and mouse data are reported (left and right columns respectively).

### Human protein validation

**Figure 4:**
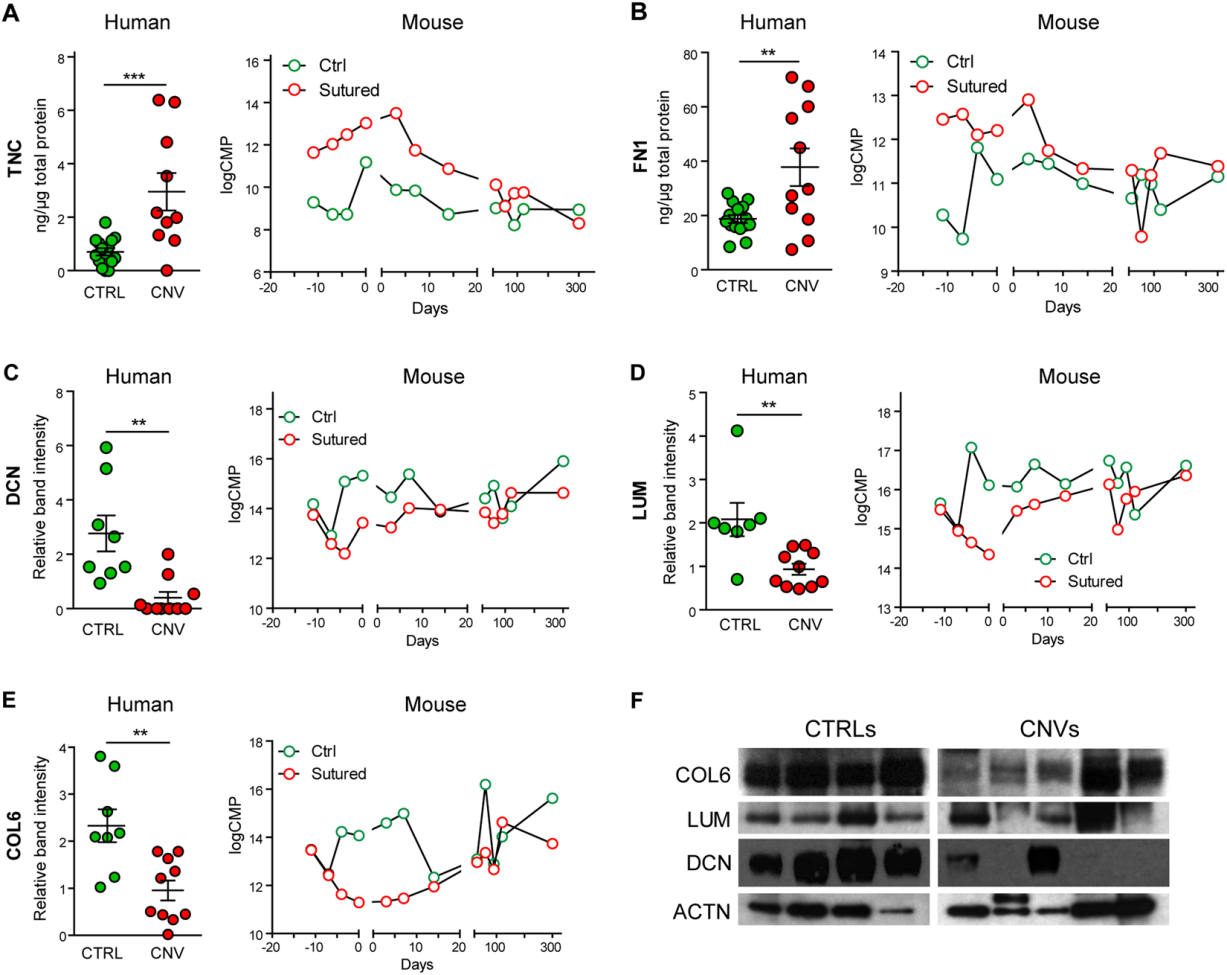
Validation of extracellular matrix proteins differentially expressed in CNV patients. On the right, mass spectrometry intensities (log CPM, Counts Per Million) for each protein in murine stroma are reported over time, while on the left the human protein level is shown as estimated by ELISA (tenascin-C and fibronectin-1, panel A and B) or Western blot (decorin, lumican and collagen-VI α-1, panels C, D and E respectively). F) Representative Western blot images cropped from different gels with different exposure. Statistical analysis performed by unpaired *t*-test (**p < 0.01, ***p < 0.001).

The differential expression of TNC, FN1, DCN, LUM and COLVI in the human corneal stroma was finally validated by WB or ELISA analysis. As shown in Fig. 4A,B, tenascin-C and fibronectin-1 levels were increased in CNV patients (+319% p = 0.0009, +102% p = 0.0047 respectively). On the other side, the expression of decorin (Fig. 4C), lumican (Fig. 4D) and collagen-VI α1 (Fig. 4E) was reduced (−85.8% p = 0.0018, −55.1% p = 0.002 and −59.1%, p = 0.0044 respectively). These data confirm the results obtained from the MS analysis of the human corneal stroma. Representative WB images are shown in Fig. 4F. For each validated protein, a graph showing the trend over time in the mouse model is reported on the right side of the corresponding panel.

## Discussion

Corneal neovascularization is a leading cause of blindness worldwide and the need for better treatment is real and urgent. To this end, the quantification of the entire human CNV proteome is a useful tool to provide novel insights into the pathophysiology of this disease, and generate novel therapeutic targets.

Until today, a few studies focused on the physiological corneal proteome^26,39–41^, but none of them analysed the pathological proteome of *human* CNV, as we did here.

In order to improve the purity and optimize ECM protein retrieval in CNV mice and humans, we took action to reduce variability. Specifically, in order to avoid unwanted dilution of stromal ECM structural proteins, and differently from previous studies^13,14^, we removed the corneal epithelium prior to tissue processing. In addition, our suture-induced CNV model allowed us to follow-up animals for a long time (10 months), with less side-effects (corneal opacity, infections and perforations) compared to other damage models used in previous studies^13,14^.

We acknowledge that pooling together murine samples can reduce variability and, hence, be a limitation of this study, although this was required for ethical reasons to limit the number of sacrificed animals. On the other hand, however, we used completely independent samples and techniques (ELISA and Western Blot) to validate our proteomics results. It should also be noted that pooling the samples makes the pool stronger in terms of stability, because the inter-individual variability is attenuated.

Cursiefen *et al*. quantified the extent of CNV growth and regression using the same murine model we used^42^, showing long-term persistence of blood vessels (up to 8 months) and total regression of lymphangiogenesis after 6 months, in the murine cornea. We were able to observe corneal blood and lymphatic vessels until 300 days after suture removal. Nonetheless, our data confirm that vessel regression continues over time, with an initial one-week, fast regression followed by a second, slower regression phase. It should be noted, however, that CNV was quantified differently in the two studies and this may have influenced the results. In fact, we performed whole-mount staining and quantified the vascular area on the entire cornea, while Cursiefen *et al*. performed quantification on corneal cross sections.

Our novel approach of CNV proteome analysis at different time points during the entire 10-month period increases the relevance of the animal model in terms of clinical translation, since patients affected with long-standing CNV are by far more numerous.

The proteomic analysis allowed us to identify, for the first time, five closely related ECM proteins, which are differentially expressed in vascularized *human* corneas.

Specifically, we found that two proteins: tenascin-C and fibronectin-1 were upregulated in the CNV cornea. Tenascin-C is highly conserved in vertebrates^43^, mostly expressed by stromal cells and leukocytes at sites of inflammation, including the cornea after refractive surgery^43–47^.

Interestingly, the increased expression of tenascin-C is maintained not only in acute, but also in chronic inflammation, where it contributes to disease severity^48^, which explains its presence in the setting of long standing CNV in our study. Tenascin-C promotes vascularization by favoring cell spreading and signaling^49,50^ and by reducing tissue rigidity^51^, which in turns promotes vascular endothelial cell invasion. Tenascin-C targeting therapies with RNA interference have been already proposed^52,53^, even in very recent studies^54^ for the treatment of glioblastoma, impairing the tumor cell migration and invasion. Our findings support a potential use of a similar TNC inhibitory approach for CNV treatment.

Fibronectin-1 is a well-known ECM protein that mediates cell adhesion, migration, growth and differentiation^55^. Of note, TNC and FN1 are expressed during the early phases of wound healing, when neovascularization is more intense^56^. Intriguingly, TNC binds to FN1^57^ and seems to regulate the pro angiogenic effects of FN1 in tumors^58^. FN1, on the other side, binds to and regulates decorin function^59^.

Infectious keratitis is a leading cause of CNV. In our study, 3 patients with CNV following Acanthamoeba infection were analyzed and we surprisingly found that they showed the highest concentration of FN1 and TNC (+116%, p = 0.044 + 49.5% p = 0.012 compared to non-Acanthamoeba CNV patients). In fact, Acanthamoeba, can bind to ocular FN1, and this is instrumental to the development of keratitis^60^. The potential role of FN1 overexpression in inducing CNV is supported by the finding that FN1 concentration is increased in contact lens wearers^61^, which are both at higher risk of developing CNV^62^ and Acanthamoeba keratitis^63^. We are aware that drawing conclusions from a population of only 3 patients is speculative: confirmation of our findings in a larger cohort of Acanthamoeba keratitis patients will be necessary, in the future, to confirm these findings.

FN1 is produced by pro-angiogenic macrophages^64^ and stimulates endothelial cell proliferation, micro-vessel elongation and angiogenesis^65^. Finally, and in line with our observation of extensive lymphangiogenesis in mice, FN1 is an excellent substrate for lymphatic endothelial cells invasion^66^, while TNC induces lymphangiogenesis in tumors^67^. Interestingly, several studies have targeted FN1 over-expression in tumors, with therapeutic molecules or even imaging agents^68,69^. This approach can be now proposed in CNV, thereby achieving *in situ* and highly-specific vascular inhibition at the site of vessel growth.

On a different note, we found that decorin and lumican were downregulated in vascularized mouse and human corneas. These proteins belong to the small leucine-rich proteoglycan family (SLRP) of the ECM^70^. They are expressed in the normal corneal stroma, where they promote corneal transparency, collagen fibril assembly and increase tissue rigidity by stabilizing collagen architecture^71^. Previous reports support the role of decorin in acute (up to 14 days) CNV in rabbit^72^. Further, it is known that deletion or mutations of DCN and LUM can lead to corneal dystrophies^73–75^. However, the relevance of decorin in *human* and in non-acute CNV has not been tested before.

It is known that DCN contributes to the maintenance of proper collagen organization^8,76^ and increases corneal stiffness^77^. The fact that DCN expression is reduced in CNV supports the concept that decreased tissue rigidity is associated with vascular invasion in the cornea, as it occurs in other tissues^24,78^. In this vein, it has been shown that increasing corneal rigidity by corneal crosslinking can inhibit CNV^79^. In addition to its effects on tissue biomechanics, overexpression of DCN retards in rabbits CNV, by down-regulating pro-angiogenic VEGF and angiopoietin^72^. In tumors, DCN-based therapies can promote tissue regeneration and reduce fibrotic scarring^80^, by downregulating several growth factors^81^.

Similarly to DCN, LUM also promotes collagen deposition, organization^82^ and stabilization^83^, therefore promoting tissue rigidity, and hence, a-vascularity. Moreover, lumican inhibits angiogenesis by interfering with α2β1 activity and downregulating MMPs expression^84^. In addition, it inhibits the influx of inflammatory cells to the cornea^85–87^, which is instrumental to CNV development^88^. Our finding confirms the results of a recent study^89^, which showed that LUM derived peptides promote corneal wound healing; this opens up a new promising field for the treatment of corneal scarring and CNV.

Finally, we observed reduced expression of collagen-VI, which is constitutively expressed in the corneal stroma^90^. Reduction in COLVI could promote CNV by altering the corneal biomechanics. For instance, COLVI depletion in the adipose tissue and tendons results in disorganized tissue morphology^91,92^. Furthermore, very recent studies reported that the C-terminal product of collagen-VI α-3, endotrophin, promotes angiogenesis and inflammation through recruitment of macrophages and endothelial cells in tumors^93^.

In addition to the five proteins whose differential expression is conserved in mouse and human subjects, we provide a list of other proteins, which are differentially expressed in mice or in human CNV samples, which will be subject of future studies.

In summary, our data globally suggest that the corneal stroma in CNV patients is highly disorganized and less rigid. These morphological modifications follow the release of soluble factors from leukocytes^94,95^, endothelial cell invasion and secretion^96^ -which also contribute to tissue rearrangement- and vascular leakage/edema, with lymphatic vessel infiltration^97^.

In conclusion, in this paper we identify, for the first time, five ECM proteins, which are conserved in humans and are differentially expressed in normal vs. vascularized corneas. We suggest that our findings may shed new light on the complex pathophysiology of human CNV, and finally lead to improved treatments.

## Supplementary information

Supplementary materials

Supplementary table S1

Supplementary table S2

Supplementary table S3
