# Supplementary material for: The role of extracellular matrix in mouse and human corneal neovascularization
Source: Sci Rep. 2019 Oct 3;9:14272. doi: 10.1038/s41598-019-50718-8 (PMC6776511; doi:10.1038/s41598-019-50718-8)
Supplement: Supplementary file 1 — Supplementary materials [file 41598_2019_50718_MOESM1_ESM.pdf]

The role of extracellular matrix in mouse and human corneal neovascularization

Barbariga M[1], Vallone F[2], Mosca E[3], Bignami F[1], Magagnotti C[2], Fonteyne P[1], Chiappori F[3], Milanesi L[3], Rama P[1], Andolfo A[2*], Ferrari G[1*].

[1]Cornea and Ocular Surface Disease Unit, Eye Repair Lab, IRCCS San Raffaele Scientific Institute, Milan, Italy. [2]ProMiFa, Protein Microsequencing Facility, IRCCS-San Raffaele Scientific Institute, Milan, Italy. [3]Institute of Biomedical Technologies, National Research Council, Segrate (MI), Italy. * Correspondence to ferrari.giulio@hsr.it; andolfo.annapaola@hsr.it.

Supplemental figure 1: comparison of identified proteins in murine and human stroma samples with the species-specific matrisome database. Venn diagram of identified proteins in mouse (panel **A** and **B**) and in human (panel **C** and **D**) compared to the matrisome-associated proteins (panel **A** and **C**) or the core-matrisome proteins (panel **B** and **D**).

Supplemental table 1: classification of murine stroma proteins accordingly to Matrisome database. Samples were compared with the matrisome-associated protein database and the core-matrisome protein database (first and second sheet respectively).

Supplemental table 2: classification of human stroma proteins accordingly to Matrisome database; samples were compared with the matrisome-associated proteins and the core-matrisome proteins (first and second sheet respectively).

Supplemental table 3: protein list from homology mapping between mouse and human corneal stroma. Only proteins differentially expressed (LFC log_2FoldChange >0.58 and <-0.69, FDR < 0.2) between sutured animals and controls and between CNV patients and controls were taken into account for the validation step.

Supplemental figure 2: whole film images of the WB lanes used in figure 4F. A) Lumican; **B**) Decorin; **C**) Actin; **D**) COLVI

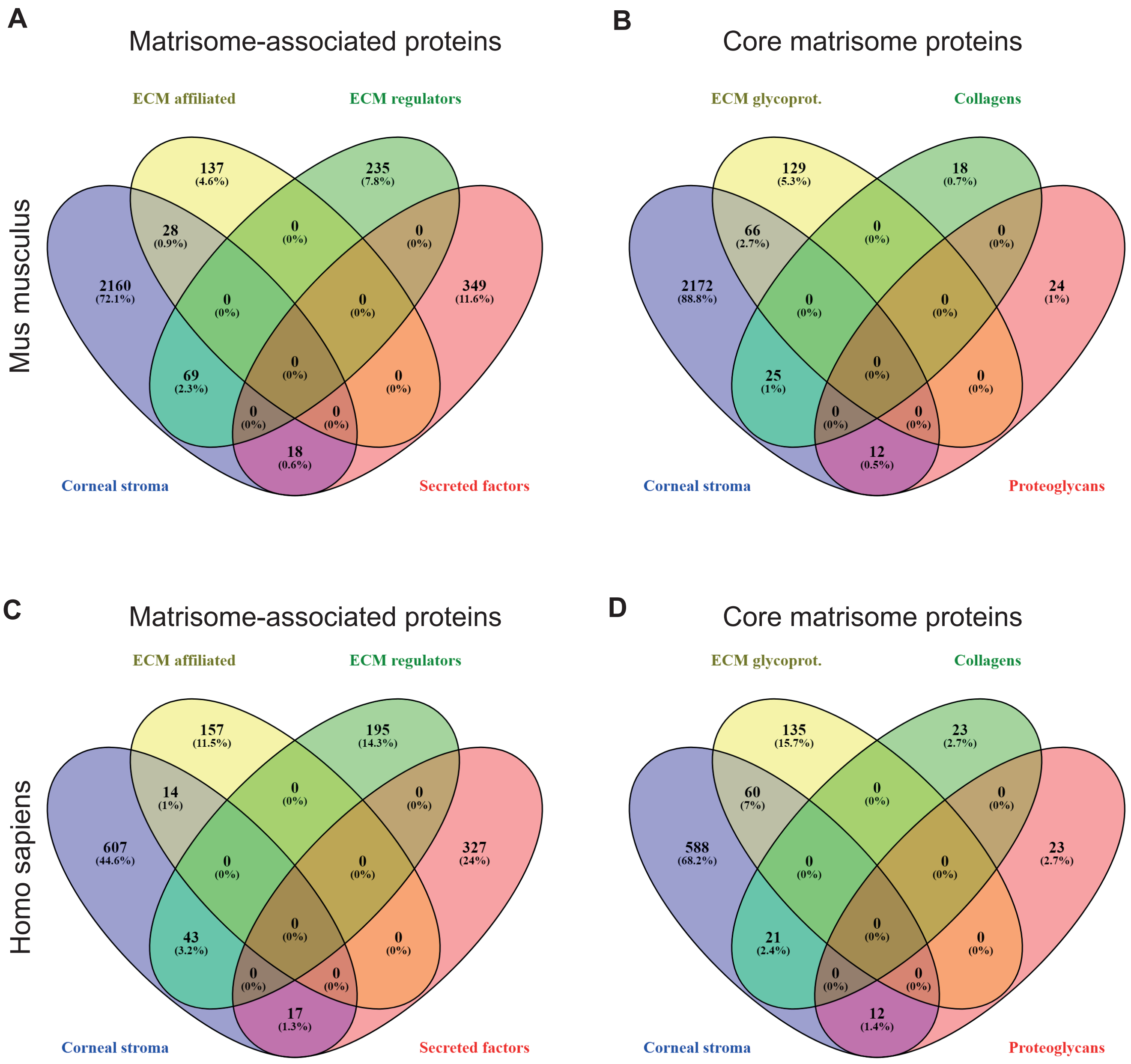
A
Matrisome-associated proteins
Mus musculus
ECM affiliated
ECM regulators
Corneal stroma
Secreted factors
137 (4.6%)
235 (7.8%)
28 (0.9%)
0 (0%)
0 (0%)
2160 (72.1%)
0 (0%)
0 (0%)
349 (11.6%)
0 (0%)
69 (2.3%)
0 (0%)
0 (0%)
0 (0%)
18 (0.6%)
B
Core matrisome proteins
ECM glycoprot.
Collagens
Corneal stroma
Proteoglycans
129 (5.3%)
18 (0.7%)
66 (2.7%)
0 (0%)
0 (0%)
2172 (88.8%)
0 (0%)
0 (0%)
24 (1%)
0 (0%)
25 (1%)
0 (0%)
0 (0%)
0 (0%)
12 (0.5%)
C
Matrisome-associated proteins
Homo sapiens
ECM affiliated
ECM regulators
Corneal stroma
Secreted factors
157 (11.5%)
195 (14.3%)
14 (1%)
0 (0%)
0 (0%)
607 (44.6%)
0 (0%)
0 (0%)
327 (24%)
0 (0%)
43 (3.2%)
0 (0%)
0 (0%)
0 (0%)
17 (1.3%)
D
Core matrisome proteins
ECM glycoprot.
Collagens
Corneal stroma
Proteoglycans
135 (15.7%)
23 (2.7%)
60 (7%)
0 (0%)
0 (0%)
588 (68.2%)
0 (0%)
0 (0%)
23 (2.7%)
0 (0%)
21 (2.4%)
0 (0%)
0 (0%)
0 (0%)
12 (1.4%)

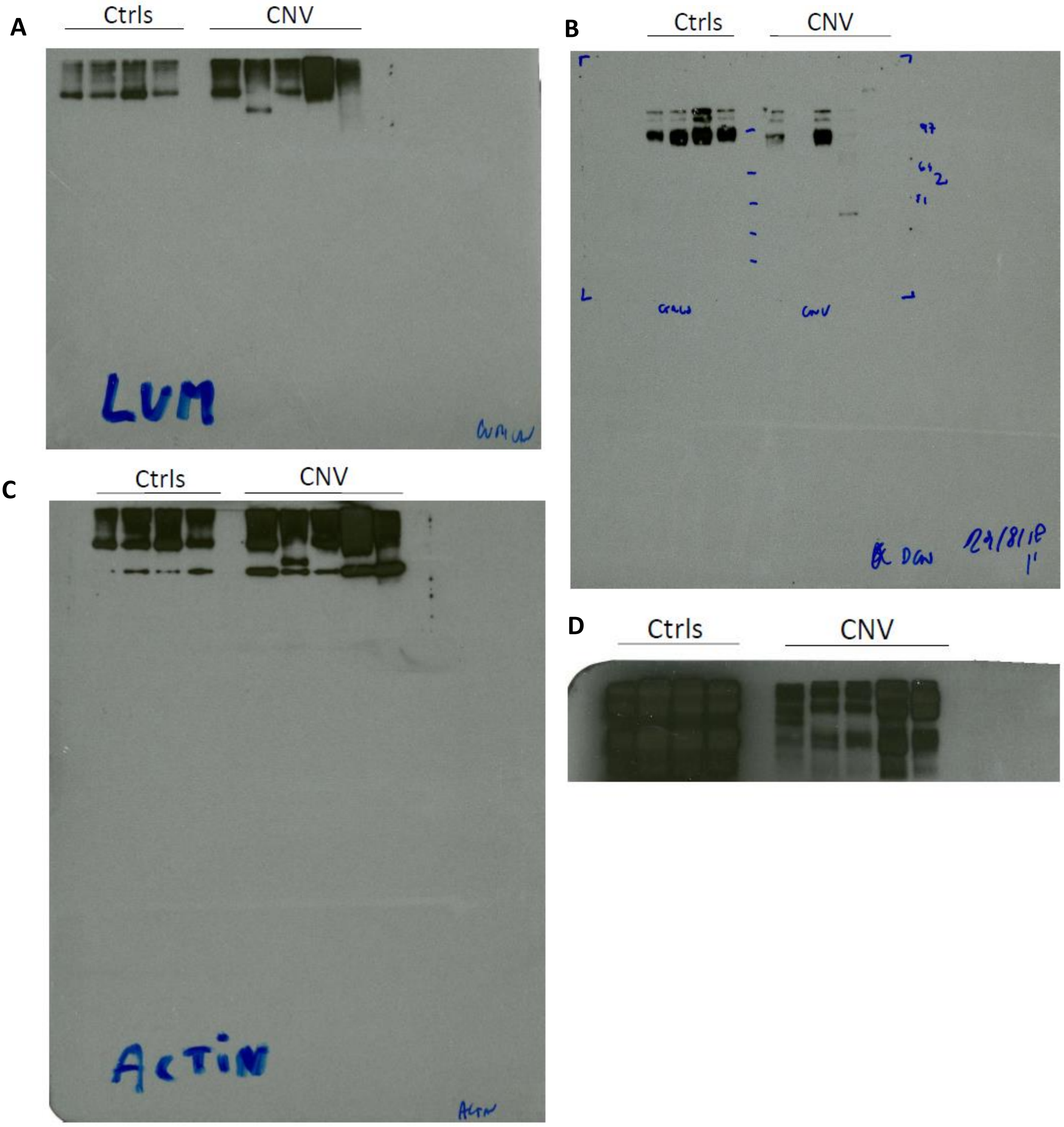

Supplemental figure 2: whole film images of the WB lanes used in figure 4F. A) Lumican; **B**) Decorin; **C**) Actin; **D**) COLVI
